# Bruxism repercussions in muscular activation: Ultrasound differences in abdominal wall between women with and without bruxism. A cross-sectional study

**DOI:** 10.1371/journal.pone.0317316

**Published:** 2025-02-07

**Authors:** Paula Yela-Lorenzo, Paola Montes-Arenas, Vanesa Abuín-Porras, Ángel González-de-la-Flor, Laura González-Fernández, Jorge Hugo Villafañe, Isabel Mínguez-Esteban

**Affiliations:** Faculty of Medicine, Department of Physiotherapy, Universidad Europea de Madrid, Health and Sports, Villaviciosa de Odón, Spain; University of Minnesota School of Dentistry, UNITED STATES OF AMERICA

## Abstract

**Introduction:**

Bruxism, often triggered by stress, induces temporomandibular alterations, increasing muscle activity and affecting dental occlusion. Prevalence of musculoskeletal conditions related to stress is higher in women, with craneo-cervical affectation being frequently reported. Moreover, some authors explore the relationship between temporomandibular disorders and postural alteration, affecting the trunk complex. This study aims to evaluate differences in abdominal muscle morphology between women with and without bruxism in Spain, and, secondarily, the effects of voluntary teeth grinding in abdominal muscle activation.

**Methodology:**

An observational analytical cross-sectional study was conducted with 44 Spanish women (diagnosed with bruxism = 22, non-bruxism = 22). To ensure and corroborate the absence of bruxism from the control group, the Clinical Based Assesment questionnaire was used. Ultrasound measures of Transverse Abdominal, External Oblique and Internal oblique were recorded during rest and voluntary grinding.

**Results:**

Grinding was associated with changes in abdominal muscle thickness in both groups, with statistically significant higher thickness values for all studied muscles. Moreover, the Bruxism group showed statistically significant higher values for all muscles in grinding conditions compared to the Non-Bruxism group. Significant differences in left transverse abdominal thickness were found between groups in resting condition. Further analysis using linear regression indicated that both Bruxism (t = -2.03, p = 0.049) and BMI (t = 3.13, p = 0.003) were significantly associated with muscle thickness, with BMI acting as a confounding factor. Age was not a significant predictor (p = 0.506), suggesting its limited role in this context.

**Conclusion:**

The results of this study support the existence of an association between bruxism and the abdominal wall muscles structure and activation. This emphasizes the importance of considering bruxism in the evaluation of abdominal muscle function.

## Introduction

Bruxism is defined as repetitive jaw-muscle activity, including teeth grinding and/or clenching or thrusting of the mandible.It can manifest in two distinct forms: sleep bruxism (occurring during sleep) and awake bruxism (occurring during wakefulness) [1]. Bruxism affects 30% of the world’s population, presenting a higher incidence and manifestation of symptoms in females [2,3]. Scientific literature often reports that women are found to experience higher levels of stress and anxiety, which are closely linked to bruxism [4].

Research consistently shows a higher prevalence of musculoskeletal disorders (MSDs) in females, with a significant association between these conditions and psychological stress, with problems in the cervical area being the most commonly reported [5–7]. Several studies reveal that prolonged stress contributes to tension in the muscles, leading to cervical myalgias and related conditions such as tension-type headaches. For example, tension-type headaches often originate from musculoskeletal impairments in the craniocervical region, where increased muscle tone and myofascial trigger points have been identified as major contributors [8,9]. Moreover, patients with chronic pain conditions like tension-type headaches show heightened stress-induced responses, which are believed to exacerbate muscular tension, leading to persistent headaches and muscle pain, especially in the neck and masticatory muscles [10]. Specifically, stress generates alterations in the orofacial region, at the cervical structure level, and in the muscles involved in the chewing process [11]. Prolonged stressful situations produce central changes, which result in neck pain and headaches, symptoms related to forced dental occlusion and teeth grinding or clenching, present in bruxism pathology [11,12].

From a structural approach, the fascia constitutes a system that indirectly connects the entire muscular structure, exerting control over body tensions, meaning that an increase in tension in one region could potentially trigger an increase in another body area [13,14]. In bruxism cases, there is an increase in muscular activity at the orofacial level, generating tensions in the cervical muscles, which affect the position of the cranial complex [15]. Through the articular system, different relationships with the cervical spine are established, forming the cranio-cervico-mandibular system [16]. The main alterations in this system are known as temporomandibular disorders (TDM), which are a potential source of postural control impairment [17]. At the mandibular level, an anterior displacement of the articular disc seems to be closely related to changes in body posture. There is some limited evidence pointing towards postural changes related to TDMs including pelvic girdle, mandibular, and spinal positions [18–22], suggesting that correct occlusion and temporomandibular position could influence spinal function [18]. The extent and the origin of this influence is yet to be determined, with a lack of evidence regarding the possible interference in the abdominal area of bruxim-related phenomena.

Ultrasound imaging (USI) is recognized as a valuable tool for assessing and quantifying the musculoskeletal and soft tissue structures. USI is an effective, safe, affordable, and easily accessible imaging modality that allows for the rapid evaluation of both morphological and pathomorphological changes in various organs and soft tissues [23–25]. Additionally, given the depth of the abdominopelvic muscles, few techniques enable the reliable, non-invasive measurement of parameters such as length, depth, diameter, cross-sectional area, volume, and rotation angles, as well as their changes and effects on related structures like fascia and organs, including the bladder and uterus, which USI is capable of measuring [24,26].

The aim of this study, that follows the line of a serie of investigations about the physiological effects of stress in women, is to assess through ultrasound measures, the differences in the morphology of the abdominal wall muscles at rest, and secondarily, under a condition of forced dental occlusion (grinding), in women aged 18 to 45 with and without bruxism in Spain.

## Materials and methods

### Study design

An observational analytical cross-sectional case-control study was designed following the STROBE criteria [27]. This study was approved by the European University of Madrid CI,240122,2024–440. Additionally, the study adheres to the bioethical norms of the Declaration of Helsinki. Before including subjects in the study, written informed consent was obtained, in which all patients accepted and showed their agreement with the project.

### Participants

A total of 72 subjects were recruited through social networks, flyers and advertising in dental clinics in Madrid, and at the European University of Madrid, iniciating the recruitment in February, 1^st^, 2024 until 31^st^ March 2024. Initially, each participant was asked to complete a questionnaire via the Google Forms platform, which allowed for a pre-selection of subjects and verification of inclusion and exclusion criteria. After reviewing these questionnaires, two study groups were established: bruxism (n = 22) and non-bruxism (n = 22). Additionally, 13 subjects were excluded from the study for not meeting the established guidelines. Inclusion criteria were: women aged 18 to 45 with and without a medical diagnosis of bruxism. The limit of 45 years was set to reduce the impact of the hormonal changes affecting stress response in the perimenopausial stage. Exclusion criteria were: a body mass index (BMI) over 31 kg/m^2^ [28], a history of abdominal surgery or hernia [29], active rheumatological conditions or connective tissue disorders [30], systemic illnesses [31], neurological disorders, neuromuscular or respiratory conditions [31,32], orthopedic surgeries involving the lumbar, pelvic, or lower limbs within the previous six months, skin conditions in the abdominal area [31] and allergy to the ultrasound gel components [33]. All participants signed an informed consent form prior to the study. In order to form the bruxism group, a certified diagnosis of bruxism from a medical or odontology professional was asked from the participants, and for the non bruxism group, the absence of symptoms was certified through a patient-reported questionnaire, the Self-Reported Bruxism Questionnaire (SBQ). The study was conducted in the facilities provided by Universidad Europea de Madrid.

For the calculation of the sample size, the G*Power 3.1.9.6 software was used, employing a statistical test of differences between two independent measures (2 groups). Based on the Transverse Abdominal thickness variation between groups of a previous similar study [34], a one-tailed hypothesis, an effect size of 0.8, an alpha error probability of 0.05, a power (1-β probability of error) of 0.8, and an allocation ratio (N2/N1) of 1 were used. This resulted in a total sample size of 44, with each group consisting of 22 subjects.

### Outcome measures

#### Bruxism

Used to establish the study groups, differentiating a group with subjects diagnosed with bruxism and another group without medical diagnoses. The absence of bruxism symptoms for this group was confirmed through the Self-Reported Bruxism Questionnaire (CBA) [34]. The CBA is a tool used to assess the presence of bruxism based on participants’ self-perception and recall of their oral habits. It consists of a series of questions designed to identify behaviors such as teeth grinding or grinding during sleep or wakefulness. Participants are typically asked to report the frequency and intensity of these behaviors, with response options often including scales for never, occasionally, frequently, and always. The questionnaire also includes items related to the associated symptoms of bruxism, such as jaw discomfort, tooth wear, or headaches.

#### Type of bruxism

Collected through medical history to identify daytime bruxism, nighttime bruxism, or both.

Ultrasound measurements: Thickness of Transverse Abdominal (TrAb), External Oblique (EO) and Internal Oblique (IO) and were recorded.

### Procedure

The study population was recruited through the Google Forms platform, considering the inclusion and exclusion criteria, resulting in a total of 44 subjects divided into two groups: "Bruxism" and "non- Bruxism." Once the sample was obtained, the initial part of the project began, consisting of completing various questionnaires to assess different aspects.

To ensure and corroborate the diagnosis of the "Non-bruxism" study group, the CBA questionnaire was used, classifying subjects into 3 subgroups: unlikely to have bruxism symptoms, clinical diagnosis with probable bruxism symptoms, and definitive bruxism symptoms. Subjects with scores less than or equal to 18 with unlikely bruxism symptoms were excluded from the "non-bruxism" group and therefore from the study.

Once the questionnaires were completed and the study groups established, an appointment was scheduled for abdominal ultrasound measurements.

#### Abdominal ultrasound

Ultrasound is a useful and effective method for evaluating the abdominal wall, offering multiple benefits. It is well-tolerated by patients, provides real-time images in different spatial planes, and can be performed in various settings.

For measuring the thickness of the TrAb, OE and OI, abdominal ultrasound was used. Subjects were placed in the supine position with hips and knees flexed at 30° and 90°, respectively, with a pillow under the head. Images were obtained using high-quality ultrasound (Sakura Sonoscape P10 model) with a linear probe (6–13 MHz frequency) in B-mode. Ultrasound gel was applied between the patient’s skin and the transducer, placed transversally to the muscle fibers at the midpoint between the eleventh rib and the iliac crest, displaced 25 mm anteromedially [24,35].

Images of 3 abdominal points were recorded following Whittaker et al [35] recommendations, obtained through palpation, which showed excellent intra and inter-rater reliability (CHF = 0.92–0.99). For the TrAb, OE and OI muscles the transducer was placed between the iliac crest and the lower edge of the rib cage, in line with the axillary midline. Image measurements were carried out using ImageJ software (version 2.0; US National Institutes of Health, Bethesda, Maryland, USA). The thickness of the abdominal wall muscles was measured between the fascial boundaries, establishing the distance between the upper and lower fascial layers of each muscle in millimeters. Each muscle was evaluated three times, and the average of the three measurements was calculated. Measurements were taken at rest and during forced dental occlusion patients were instructed to “grind your teeth as hard as you could” during the latest measurement condition.

### Data analysis

For statistical analysis of the obtained data, the R-Based Jamovi V.2.3 program was used. All data were analyzed with a 95% confidence interval (CI) (error α of 0.05) and a desired power of 80% (error β of 0.2). Normality for each variable was assessed using the Shapiro-Wilk test due to the sample size being less than 50 subjects, selecting parametric or non-parametric tests accordingly. Descriptive statistics were initially computed for the entire sample and for both groups individually. Measures of central tendency and dispersion were used, with the mean and standard deviation (SD) reported for parametric data, and the median and interquartile range (IR) used for non-parametric data. A comparative analysis was then conducted between the bruxism group and the non-bruxism group. Parametric data were assessed using the independent samples t-test, while non-parametric data were analyzed with the Mann-Whitney U-test. Additionally, Levene’s test was applied to assess variance equality. Additionally, a four-level intra-participant factor (occlusion and NO occlusion; right and left sides) was considered for these variables. Given the two distinct groups measured four times and the sample size, a repeated measures ANOVA design was chosen for statistical analysis. Finally, correlation between variables was analyzed with Spearman’s test, with the following interpretation of rank values: a correlation coefficient (rho) between 0.00 and 0.19 was considered very weak, between 0.20 and 0.39 as weak, between 0.40 and 0.59 as moderate, between 0.60 and 0.79 as strong, and between 0.80 and 1.0 as very strong.

## Results

The total sample description and according to the study group is presented in Table 1 along with descriptive variables. The bruxism group included a total of 6 subjects with nighttime bruxism, 2 subjects with daytime bruxism, and 14 subjects with both types of bruxism. In the descriptive analysis of demographic variables, no statistically significant results were obtained between the bruxism and non-bruxism groups, with p-value (p > 0.05), indicating that the groups were well-matched for these demographic variables. However, significant differences were observed in the left transverse abdominal (TrAb) thickness between the two groups at rest (p = 0.042).

**Table 1:** Demographic data and Outcome measures at rest condition.

Outcomes	BRUXISM (n = 22)	NON -BRUXISM (n = 22)	*P—*Value
Age	27,0 (20,75) †	25,0 (11,25) †	0.190‡
BMI	23,9 (3,06) *	22,0 (5,68) †	0.275‡
Mean Left Transversus Abdominis (mm)	2,73 (0,627) †	3,12 (0,843)*	0.042‡
Mean Right Transversus Abdominis(mm)	2,644 (0,578)*	2,76 (0,743)†	0.218‡
Mean Left Internal Oblique(mm)	6,304 (1,539)*	7,132 (1,630)*	0.091**
Mean Right Internal Oblique(mm)	6,789 (1,497)*	6,95 (1,529) †	0.481‡
Mean Left External Oblique(mm)	5,458 (1,437)*	5,27 (1,711) †	0.860‡
Mean Right External Oblique(mm)	5,537 (1,323)*	5,957 (1,909)*	0.401**

Abbreviations: mm; milimeters

* Data expressed as mean (standard deviation)

† Data expressed as median (interquartile range)

** Student’s t-test for independent samples was performed

‡ Mann-Whitney U test was performed; x Chi-square (X^2^) test was performed.

**Table 2** provides a comparative analysis of muscle thickness under resting and grinding conditions for both groups. In the grinding condition, statistically significant increases in muscle thickness were observed across all muscles studied (TrAb, IO and EO) in the studied sample (p < 0.001). This increase suggests that voluntary teeth grinding leads to higher recruitment of the abdominal muscles, which was more pronounced in the bruxism group. Specifically, the significant group-by-condition interaction effects (F-values and p-values reported) highlight that the bruxism group demonstrated greater changes in muscle thickness compared to the non-bruxism group.

**Table 2:** Comparison between conditions.

	Rest	Grinding	Condition	Group X Condition
ResUlts (n)	Mean (SD)	Mean (SD)	F (Df); p; η^2^	F (Df); p; η^2^
TrAbd R				
Non Bruxism	2,983 (0,9663)	2,908 (0,9964)	F(1) = 24,0; **p<0.001**;0,014	F(1) = 46,2; **p<0.001**; 0,027
Bruxism	2,644 (0,5780)	3,104 (0,6347)		
TrAbd L				
Non Bruxism	3,118 (0,8426)	3,063 (0,8490)	F(1) = 20,6; **p<0.001**;0,022	F(1) = 32,0; **p<0.001**;0,034
Bruxism	2,777 (0,5826)	3,271 (0,6276)		
IOR				
Non Bruxism	7,398 (2,1480)	7,371 (2,2017)	F(1) = 11,3; **p = 0.002**; 0.003	F(1) = 14,8; **p<0.001**; 0.003
Bruxism	6,789 (1,4966)	7,197 (1,5049)		
IOL				
Non Bruxism	7,132 (1,6303)	7,060 (1,6942)	F(1) = 9,33; **p = 0.004**; 0.005	F(1) = 16,26; **p<0.001**; 0.009
Bruxism	6,304 (1,5393)	6,823 (1,4749)		
EOR				
Non Bruxism	5,957 (1,9086)	5,889 (2,0094)	F(1) = 5,98; **p = 0.019**;0,001	F(1) = 14,42; **p<0.001**;0.003
Bruxism	5,537 (1,3230)	5,848 (1,3323)		
EOL				
Non Bruxism	5,575 (1,5317)	5,523 (1,6234)	F(1) = 5,69; **p = 0.022**;0,002	F(1) = 11,71; **p = 0.001**;0,003
Bruxism	5,458 (1,4369)	5,748(1.3784)		

Abbreviations: SD (standard deviation); Df (degrees of freedom); Tr Abd, Ttransverse Abdominal); IO (internal Oblique); EO (External Oblique); L (left); R (right). All thickness values are expressed in milimetres. ANOVA for repeated measures was used for the statistical analysis.

To further evaluate the relationship between Bruxism and muscle thickness while considering potential confounding factors, we conducted a linear regression analysis with Bruxism, BMI, and Age as predictors. The model demonstrated that Bruxism was significantly associated with the Mean Transverse Abdominis Thickness (t = -2.03, p = 0.049), supporting our hypothesis that Bruxism is associated with muscle morphology. While BMI was also a significant predictor (t = 3.13, p = 0.003), this likely reflects its general association with body composition rather than a specific confounding effect. In our sample, the distribution of BMI categories was as follows: 63.6% of participants had a normal BMI, 20.5% were classified as overweight, 6.8% as underweight, 6.8% fell into the Obesity I category, and 2.3% were classified as Obesity III. The majority of the sample (nearly two-thirds) had a normal BMI, indicating limited variability in body composition across the study population. This supports the interpretation that BMI is unlikely to be a major confounder in the observed relationship between Bruxism and muscle thickness.Age was not a significant predictor (t = 0.67, p = 0.506), suggesting that it does not interfere in the relationship between Bruxism and muscle thickness.

Regarding the internal oblique, significant differences were found between the two study groups, with p-values (p<0.001) for both the right and left internal oblique muscles. P-values of (p = 0.002) and (p = 0.004) were also obtained for the right and left internal oblique muscles, respectively, when comparing the rest position and forced dental occlusion, revealing statistically significant differences at this level.

Statistically significant differences were concluded for the external oblique muscle thickness between the bruxism and non-bruxism groups, with a p-value (p<0.001) for both the right and left sides. P-values of (p = 0.019) for the right external oblique and (p = 0.022) for the left external oblique showed differences between the rest position and forced dental occlusion of this muscle.

In the correlation analysis, regarding the transverse abdominal muscle, moderate correlations were observed between the right and left transverse muscles at rest, with p = 0.001 and Rho = 0.472, and between the right and left transverse muscles during forced dental occlusion, with p = 0.001 and Rho = 0.465.

For the internal and external obliques, strong correlations were found in both cases, with p-values of p<0.001 and Rho = 0.677 for the right and left internal obliques at rest; p<0.001 and Rho = 0.575 for the right and left external obliques at rest; p<0.001 and Rho = 0.677 for the right and left internal obliques during forced dental occlusion; and p<0.001 and Rho = 0.655 for the right and left external obliques during forced dental occlusion.

Additionally, statistically significant correlations were analyzed between different muscles on the same side (right or left) in the same condition (rest or forced dental occlusion).

On the left abdominal wall, strong correlations were found between the transverse and internal oblique muscles in the resting position, with p<0.001 and Rho = 0.561, and in the occlusion condition, with p<0.001 and Rho = 0.646. Strong correlations were also established between the internal and external obliques in the resting condition, with p<0.001 and Rho = 0.520, and during forced dental occlusion, with p<0.001 and Rho = 0.576.

For the right abdominal wall, no statistically significant correlations were observed with the transverse abdominal muscle, with a p-value (p>0.05); however, strong correlations were found between the internal and external obliques in the resting condition, with p<0.001 and Rho = 0.544, and during forced dental occlusion, with p<0.001 and Rho = 0.631.

## Discussion

The results of this study show a statistically significant relationship between the presence bruxism and the thickness of some abdominal wall muscles (TrAbL) compared to non-bruxism. Moreover, these significant differences between the two study groups were extended under grinding condition to all the analyzed abdominal wall muscles (TrAb, IO and EO), with higher values in the women with bruxism. No significant differences were observed in BMI (p = 0.275) or Age (p = 0.199) between the Bruxism and non-bruxism groups. These non-significant results suggest that neither BMI nor Age systematically varies with Bruxism status, minimizing their potential roles as confounding factors in this context. Although BMI was significant in the regression analysis, this is likely due to its general association with muscle size rather than a direct influence on the observed group differences. The lack of significance for Age further supports the notion that age-related changes do not account for the differences in muscle thickness associated with Bruxism.

From a muscle activation perspective, efforts involving core stabilization result in increased tone of masticatory muscles. In the study by Moon et al.[36], it was shown that co-contraction of masticatory muscles during neck stabilization exercises significantly increases the thickness of the longus colli muscle, suggesting that central stabilization influences masticatory muscle activity. The results of this study allow hypothesizing that overactivation of masticatory muscles could impact core muscle activation, leading to greater recruitment and thus thicker ultrasound images. Several authors relate jaw grinding to physiological phenomena such as enhanced activity, force production, and joint fixation [37,38]. This preactivation of muscles has been widely studied in neurology and motor imagery but not in bruxism and core muscles.

Moreover, the findings of Chu et al. [39] are in line with research suggesting that psychological stress is a significant factor in the onset and exacerbation of musculoskeletal disorders, particularly those involving the temporomandibular joint. It is hypothesized that stress-related hyperactivation of the masticatory muscles, compounded by repetitive jaw movements such as bruxism, could lead to a chronic increase in muscle tone and a subsequent elevation in the baseline muscle activity of both masticatory and core muscles, potentially explaining the observed correlations between bruxism, TMD, and core stabilization efforts [40,41].

A study by Staniszewski et al. [42] suggests that patients with TMD may have an upregulated Hypothalamic-Pituitary-Adrenal) axis with higher glucocorticoid secretion, indicating a connection between psychological factors and dysfunction of the temporomandibular joint and masticatory muscles.

From a structural point of view, the fascial system creates synergy between different body structures, allowing connections between all body parts. According to this model, the presence of a caudal mechanical pressure exerted by sustained forced dental occlusion in bruxism, reflected at the abdominal level, could generate an increase in pressure in this area [43], which could offer an structural explanation for the observed increase in the thickness of all abdominal muscles under forced dental occlusion in this study, with a more pronounced increase in the bruxism group.

According to a study by Xiaode Liu et al. [44], sustained pressure at the abdominal level triggers an increase in intraabdominal pressure. In response to sustained intra-abdominal pressure mainly due to sustained contraction of orofacial muscles, prevalent in bruxist populations, it is plausible that the abdominal wall might respond with prolonged contraction, leading to increased muscle tone and therefore greater thickness in this area. This rationale could potentially explain the increased thickness of the left transverse abdominal muscle at rest in the bruxism group compared to the non-bruxism group.

Most of the examined muscles showed positive correlations between both sides of the body them regarding thickness. However, in the right abdominal wall, only a relationship between the internal and external oblique muscles was observed, excluding the transverse abdominal muscle from any association. Differences in the abdominal wall due to laterality or sports practice have been studied using ultrasound by various authors [28,45,46].

Several non-significant findings were observed in this study. Notably, the right transverse abdominis thickness did not differ significantly between the Bruxism and Non-Bruxism groups, contrasting with the significant findings for the left side. This may indicate a potential laterality effect, possibly influenced by individual differences in muscle activation, which warrants further investigation [47–50]. Additionally, no significant differences were found for the internal oblique muscles and external oblique muscles suggesting that the association of bruxism with muscle thicknessmay be more pronounced in deeper core muscles like the transverse abdominis.

The findings of this study have various implications for clinical practice, especially in managing patients with bruxism and core stability-related problems. Using USI to assess abdominal wall thickness can be a useful tool for identifying significant differences in bruxist patients and promote tailored interventions that include stress management techniques. Future research lines may include extrapolations of the results of the present study to male population. In addition, electromyographic data recording the activation of abdominal muscles during grinding in population affected with bruxism could give a more accurate picture of the relationship between the two structures.

### Limitations

One of the key limitations of this study is the relatively small sample size, which may limit the generalizability of the findings. The CBA was chosen due to its non-invasive and practical application in a clinical setting. However, we acknowledge that it may not fully capture the severity of bruxism. Participants in the bruxism group were asked to bring a certificated diagnosis from either medical or odontology professionals, although the tests that have driven these professionals to the diagnosis (electromyographic studies or polysomnograms) were not recorded. Additionally, the study only included women, which restricts the applicability of the results to male populations. Another limitation is the absence of longitudinal data; since this was a cross-sectional study, it is not possible to infer causality between bruxism and abdominal muscle thickness further than an hiphotesis testing. A potential limitation of our study is the effect of BMI as a covariate, which may not fully capture the complexities of body composition, such as muscle mass or fat distribution. Although BMI was statistically significant in our model, its role appears more related to general body size rather than specific effects on the relationship between Bruxism and muscle thickness. Future studies should consider more precise measures of body composition to further validate these findings. Furthermore, while ultrasound imaging is a reliable method for measuring muscle thickness, it does not capture the functional aspects of muscle activity, such as contraction strength or endurance. Lastly, the lack of electromyographic data limits the ability to correlate ultrasound findings with muscle activation patterns during grinding, which could provide a more comprehensive understanding of the bruxism-muscle relationship.

## Conclusion

The findings of this study show differences in the thickness of abdominal muscles under rest and voluntary grinding conditions, suggesting a relationship between the activation of the two areas, in addition to the fact that there were also statistically significant differences for all the studied muscles in the two study groups, bruxism and non-bruxism, during the grinding condition. The results also showed. certain association of bruxism with muscle thickness of the Transverse Abdominis. The significant result from the linear regression analysis indicates that Bruxism is associated with muscle morphology independently of BMI and Age. However, while BMI was a significant predictor this likely reflects general body composition rather than a specific confounding effect. Age did not significantly affect the outcome. These results align with the proposed physiological mechanism, suggesting that repetitive jaw activity in Bruxism contributes to altered core muscle recruitment. Nevertheless, given the cross-sectional nature of the study, causality cannot be inferred, and further longitudinal studies are needed to confirm these associations.

## Supporting information

S1 File Database bruxism. Complete Database Anonimyzed.
(XLSX)
