# Correction: Bruxism repercussions in muscular activation: Ultrasound differences in abdominal wall between women with and without bruxism: A cross-sectional study

**DOI:** 10.1371/journal.pone.0321772

**Published:** 2025-04-01

**Authors:** Paula Yela-Lorenzo, Paola Montes-Arenas, Vanesa Abuín-Porras, Ángel González-de-la-Flor, Laura González-Fernández, Jorge Hugo Villafañe, Isabel Mínguez-Esteban

There is an error in the affiliation of authors. The correct affiliation is: Universidad Europea de Madrid, Department of Physiotherapy, Faculty of Medicine, Health and Sports, Villaviciosa de Odón, Spain.
